# Supplementary material for: Using smart speakers to contactlessly monitor heart rhythms
Source: Commun Biol. 2021 Mar 9;4:319. doi: 10.1038/s42003-021-01824-9 (PMC7943557; doi:10.1038/s42003-021-01824-9)
Supplement: Supplementary file 2 — Supplementary Information [file 42003_2021_1824_MOESM2_ESM.pdf]

Using Smart Speakers to Contactlessly Monitor Heart Rhythms

Supplementary Materials

Anran Wang, Dan Nguyen, Arun R Sridhar, Shyamnath Gollakota

Age	31.0 (IQR: 8.5) years
BMI	22 (IQR: 3)
Female to male ratio	0.6

Supplementary Table 1: Demographic summary (median and interquartile range) of cohort of 26 healthy participants.

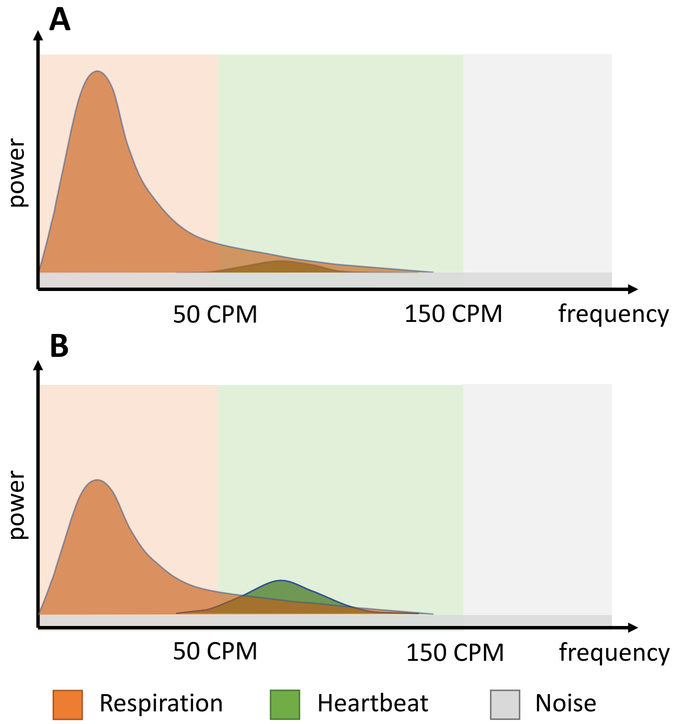

Supplementary Figure 1: Illustrative signals in the frequency domain (**A**) before beamforming; and (**B**) after beamforming. The residual interference from respiration after beamforming is not negligible.

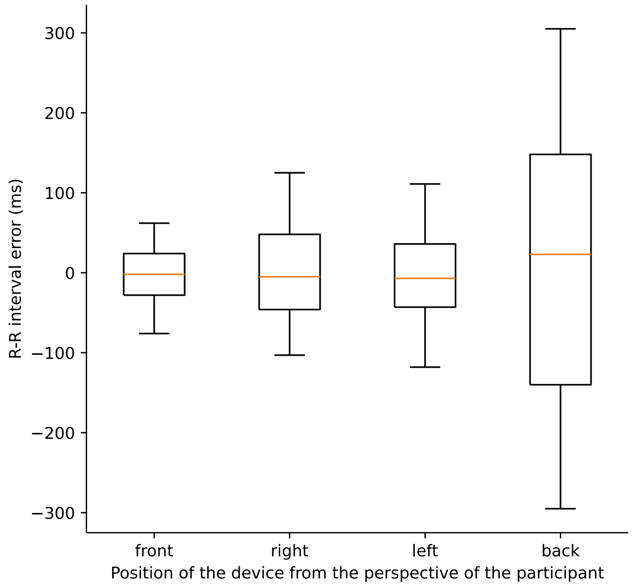

Supplementary Figure 2: The 95-percentile, 75-percentile, median, 25-percentile and 5-percentile R-R interval error when the device is placed at the front, towards the sides and the back of the participant (n = 4) at a distance of 40 cm.

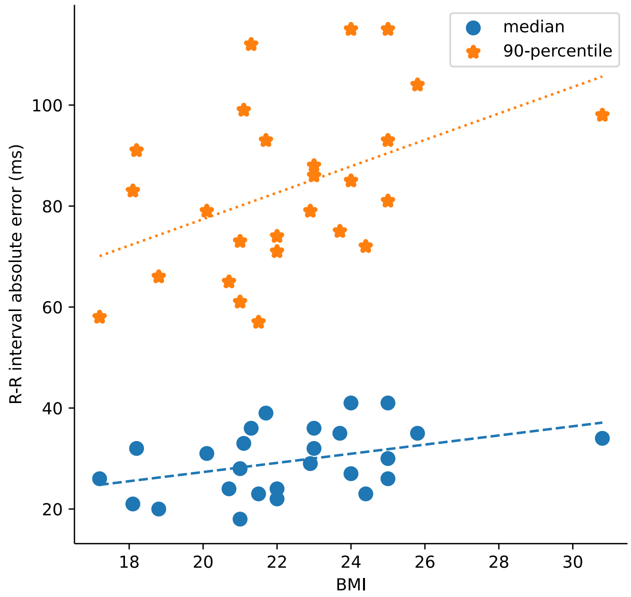

Supplementary Figure 3: The relationship between BMI and R-R interval errors for each of the healthy participants.

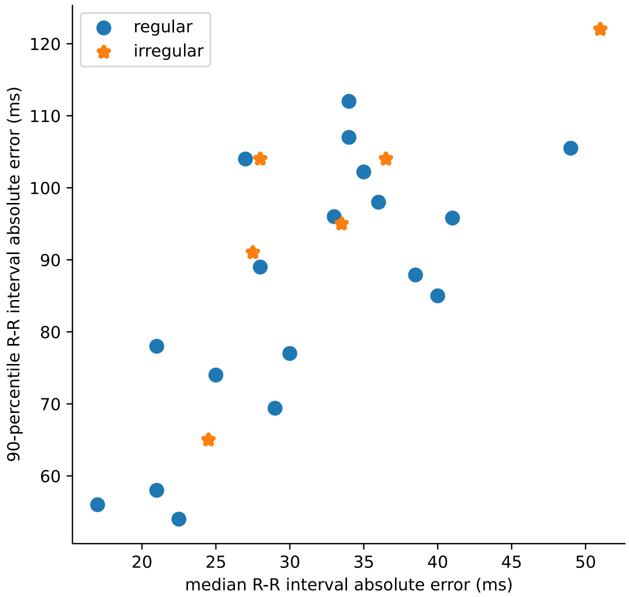

Supplementary Figure 4: The R-R interval absolute errors for each individual with regular and irregular rhythms among the hospitalized participants.

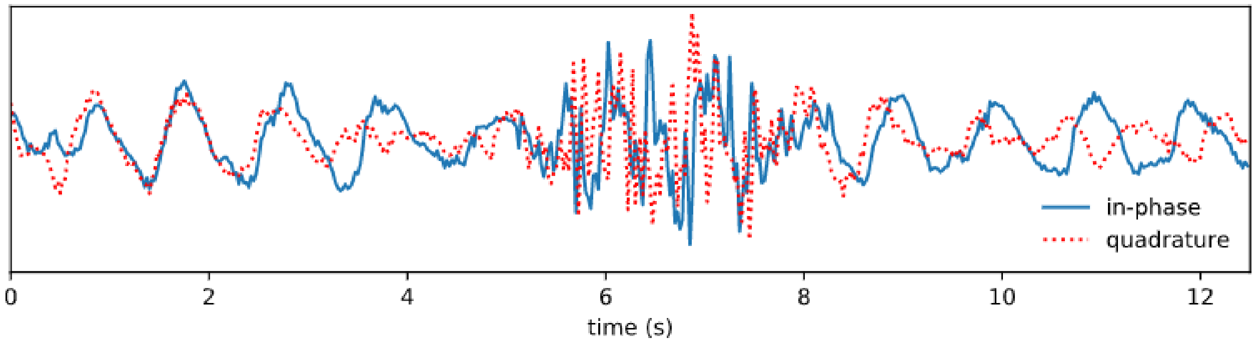

Supplementary Figure 5: Heart rhythm signal with gross body motion. A period of the in-phase and quadrature phase acoustic signals corresponding to heart rhythm. The signal is affected around the sixth second is affected by arm movements by the participant.

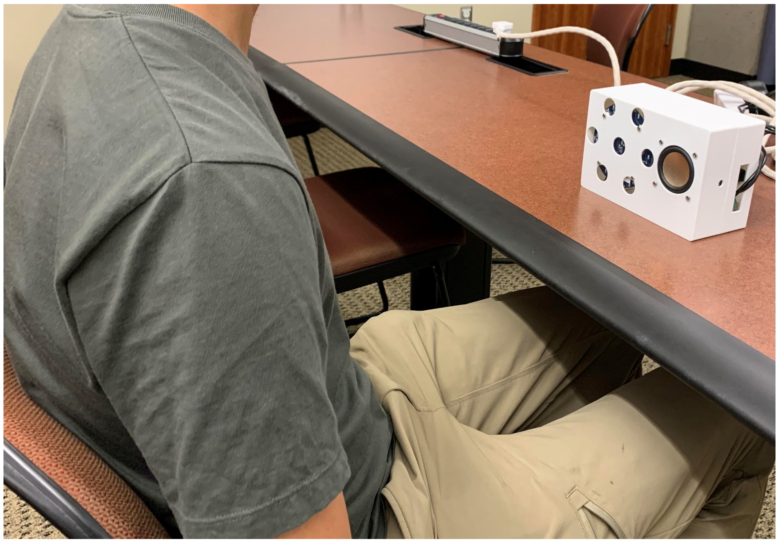

Supplementary Figure 6: Smart speaker prototype and experimental setup.

Input : s: signal after filtering, R: block sampling rate (block per minute)
Output: P where P_i points to the starting point of the segment ending in i

$R_H = R/50, R_L = R/150$ {minimum and maximum segment length}
$l = len(s)$
$N_{DP}[l, R_H] = 0, DP[l, R_H] = 0, prev[l, R_H] = 0$
for i *in* $[R_H..l]$ **do**
  for j *in* $[R_H..R_L]$ **do**
    $x = s[i - j..i]$
    $k_{min} = 0$, $d_{min} = \infty$
    for k *in* $[R_H..R_L]$ **do**
      $y = s[i - j - k..i - j]$
      Resample x, y to the same length of the their longer using linear interpolation
      $x = \sqrt{\frac{x^*y}{y^*x}}x$
      $d = \frac{||x-y||_2^2}{||x+y||_2^2}$
      if $d < d_{min}$ **then**
        $d_{min} = d$, $k_{min} = k$
      end
    end
    $prev[i, j] = k_{min}$
    $DP[i, j] = DP[i - j, k_{min}] + d_{min}$
    $N_{DP}[i, j] = N_{DP}[i - j, k_{min}] + 1$
  end
end
for i *in* $R_H * 2..l - R_L$ **do**
  $j_{min} = -1$
  for j *in* $R_L..R_H$ **do**
    if $j_{min} < 0$ *or* $\frac{DP[i+j,j]}{N_{DP}[i+j,j]} < \frac{DP[i+j_{min},j_{min}]}{N_{DP}[i+j_{min},j_{min}]}$ **then**
      $j_{min} = j$
    end
  end
  $P[i] = prev[i + j_{min}, j_{min}]$
end
return P

Supplementary Algorithm 1: The heartbeat segmentation algorithm.
