# Supplementary material for: Using smart speakers to contactlessly monitor heart rhythms
Source: Commun Biol. 2021 Mar 9;4:319. doi: 10.1038/s42003-021-01824-9 (PMC7943557; doi:10.1038/s42003-021-01824-9)
Supplement: Supplementary file 3 — Description of Additional Supplementary Files [file 42003_2021_1824_MOESM3_ESM.pdf]

Description of Additional Supplementary Files

File name: Supplementary Data 1

Description: R-R intervals and heart rate data for healthy participants

File name: Supplementary Data 2

Description: R-R intervals and heart rate data for hospitalized participants

File name: Supplementary Data 3

Description: R-R intervals under different conditions.
