# Supplementary material for: Using smart speakers to contactlessly monitor heart rhythms
Source: Commun Biol. 2021 Mar 9;4:319. doi: 10.1038/s42003-021-01824-9 (PMC7943557; doi:10.1038/s42003-021-01824-9)
Supplement: Supplementary file 7 — Reporting Summary [file 42003_2021_1824_MOESM7_ESM.pdf]

Corresponding author(s): Anran Wang, Dan Nguyen, Arun Sridhar, Shyamnath Gollakota

Last updated by author(s): Dec 29, 2020

Reporting Summary

Nature Research wishes to improve the reproducibility of the work that we publish. This form provides structure for consistency and transparency in reporting. For further information on Nature Research policies, see our Editorial Policies and the Editorial Policy Checklist.

Statistics

For all statistical analyses, confirm that the following items are present in the figure legend, table legend, main text, or Methods section.

n/a	Confirmed	
☐	☒	The exact sample size (*n*) for each experimental group/condition, given as a discrete number and unit of measurement
☐	☒	A statement on whether measurements were taken from distinct samples or whether the same sample was measured repeatedly
☐	☒	The statistical test(s) used AND whether they are one- or two-sided<br>*Only common tests should be described solely by name; describe more complex techniques in the Methods section.*
☐	☒	A description of all covariates tested
☐	☒	A description of any assumptions or corrections, such as tests of normality and adjustment for multiple comparisons
☐	☒	A full description of the statistical parameters including central tendency (e.g. means) or other basic estimates (e.g. regression coefficient) AND variation (e.g. standard deviation) or associated estimates of uncertainty (e.g. confidence intervals)
☐	☒	For null hypothesis testing, the test statistic (e.g. F, t, r) with confidence intervals, effect sizes, degrees of freedom and P value noted<br>*Give P values as exact values whenever suitable.*
☐	☒	For Bayesian analysis, information on the choice of priors and Markov chain Monte Carlo settings
☐	☒	For hierarchical and complex designs, identification of the appropriate level for tests and full reporting of outcomes
☐	☒	Estimates of effect sizes (e.g. Cohen's *d*, Pearson's *r*), indicating how they were calculated

Our web collection on statistics for biologists contains articles on many of the points above.

Software and code

Policy information about availability of computer code

Data collection	We wrote custom C++ code using generic audio drivers. Code is available upon request with a non-commercial license.
Data analysis	We wrote custom Python code using common open-source libraries such as Numpy and Pytorch. Code is available upon request with a non-commercial license.

For manuscripts utilizing custom algorithms or software that are central to the research but not yet described in published literature, software must be made available to editors and reviewers. We strongly encourage code deposition in a community repository (e.g. GitHub). See the Nature Research guidelines for submitting code & software for further information.

Data

Policy information about availability of data

All manuscripts must include a data availability statement. This statement should provide the following information, where applicable:

- Accession codes, unique identifiers, or web links for publicly available datasets
- A list of figures that have associated raw data
- A description of any restrictions on data availability

All data required to interpret the results is included in the manuscript. Supplementary Data 1 and 2 includes raw data points to produce Figure 3 and 5.

Field-specific reporting

Life sciences study design

All studies must disclose on these points even when the disclosure is negative.

Sample size	26 healthy participants and 29 hospitalized patients
Data exclusions	We excluded 5 extreme obese hospitalized patients for our main study.
Replication	Replication should be successful if following the same hardware, algorithm and experimental setup.
Randomization	Randomization was not applicable to our study since we do not allocate participant into different groups randomly.
Blinding	Investigators were not blinded to group allocation because the locations of data collection for healthy participants and hospitalized patients were different.

Reporting for specific materials, systems and methods

We require information from authors about some types of materials, experimental systems and methods used in many studies. Here, indicate whether each material, system or method listed is relevant to your study. If you are not sure if a list item applies to your research, read the appropriate section before selecting a response.

Materials & experimental systems

n/a	Involved in the study	
☒	☐	Antibodies
☒	☐	Eukaryotic cell lines
☒	☐	Palaeontology and archaeology
☒	☐	Animals and other organisms
☐	☒	Human research participants
☒	☐	Clinical data
☒	☐	Dual use research of concern

Methods

n/a	Involved in the study	
☒	☐	ChIP-seq
☒	☐	Flow cytometry
☒	☐	MRI-based neuroimaging

Human research participants

Policy information about studies involving human research participants

Population characteristics	Healthy participants: age: 31(IQR: 8.5) years; BMI: 22(IQR: 3); Female-to-male ratio: 0.6.<br>Hospitalized participants: see Table 1 in the manuscript for detail.
Recruitment	We recruited a cohort of 26 voluntary participants who had no prior history of cardiac conditions. Most of them are friends, coworkers or relatives of the authors. We enrolled hospitalized cardiac patients in a medical center with their consent.
Ethics oversight	This study was approved by the University of Washington Institutional Review Board.

Note that full information on the approval of the study protocol must also be provided in the manuscript.
